# (*E*)-4-Nitro-*N*′-(3-nitro­benzyl­idene)benzohydrazide

**DOI:** 10.1107/S1600536812004540

**Published:** 2012-02-10

**Authors:** Xiao-Yan Li

**Affiliations:** a Zibo Vocational Institute, Zibo 255314, People’s Republic of China

## Abstract

The title compound, C_14_H_10_N_4_O_5_, has an *E* conformation with respect to the C=N bond. The dihedral angle between the benzene rings is 2.41 (14)°. In the crystal, mol­ecules are linked through N—H⋯O hydrogen bonds to form chains along the *c* axis. C—H⋯O inter­actions are also present, linking the chains to form a three-dimensional network.

## Related literature

For the syntheses and crystal structures of hydrazone compounds, see: Hashemian *et al.* (2011 ▶); Lei (2011 ▶); Shalash *et al.* (2010 ▶). For the crystal structures of similar compounds, reported on by the author, see: Li (2011*a* ▶,*b* ▶, 2012 ▶).
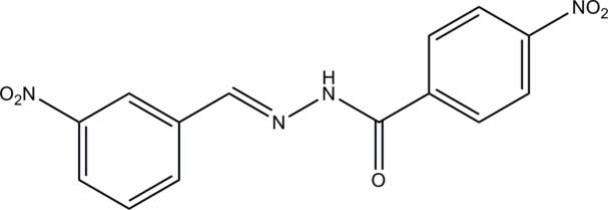


## Experimental

### 

#### Crystal data

C_14_H_10_N_4_O_5_
*M* *_r_* = 314.26
Monoclinic, 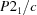

*a* = 11.856 (2) Å
*b* = 14.116 (3) Å
*c* = 8.6263 (19) Å
β = 95.193 (2)°
*V* = 1437.8 (5) Å^3^
*Z* = 4
Mo *K*α radiation
μ = 0.11 mm^−1^
*T* = 298 K
0.17 × 0.13 × 0.12 mm

#### Data collection

Bruker SMART CCD area-detector diffractometer
Absorption correction: multi-scan (*SADABS*; Sheldrick, 1996 ▶) *T*_min_ = 0.981, *T*_max_ = 0.987
10319 measured reflections
2671 independent reflections
1288 reflections with *I* > 2σ(*I*)
*R*_int_ = 0.104

#### Refinement

*R*[*F* ^2^ > 2σ(*F* ^2^)] = 0.052
*wR*(*F* ^2^) = 0.123
*S* = 0.84
2671 reflections
211 parameters
1 restraint
H atoms treated by a mixture of independent and constrained refinement
Δρ_max_ = 0.18 e Å^−3^
Δρ_min_ = −0.16 e Å^−3^

### 

Data collection: *SMART* (Bruker, 1998 ▶); cell refinement: *SAINT* (Bruker, 1998 ▶); data reduction: *SAINT*; program(s) used to solve structure: *SHELXS97* (Sheldrick, 2008 ▶); program(s) used to refine structure: *SHELXL97* (Sheldrick, 2008 ▶); molecular graphics: *SHELXTL* (Sheldrick, 2008 ▶); software used to prepare material for publication: *SHELXTL*.

## Supplementary Material

Crystal structure: contains datablock(s) global, I. DOI: 10.1107/S1600536812004540/su2375sup1.cif

Structure factors: contains datablock(s) I. DOI: 10.1107/S1600536812004540/su2375Isup2.hkl

Supplementary material file. DOI: 10.1107/S1600536812004540/su2375Isup3.cml

Additional supplementary materials:  crystallographic information; 3D view; checkCIF report

## Figures and Tables

**Table 1:** Hydrogen-bond geometry (Å, °)

*D*—H⋯*A*	*D*—H	H⋯*A*	*D*⋯*A*	*D*—H⋯*A*
N2—H2*A*⋯O3^i^	0.91 (2)	1.99 (2)	2.853 (3)	158 (2)
C6—H6⋯O1^ii^	0.93	2.57	3.369 (4)	145
C7—H7⋯O5^iii^	0.93	2.56	3.287 (4)	135
C7—H7⋯O3^i^	0.93	2.53	3.271 (3)	137
C14—H14⋯O4^iii^	0.93	2.40	3.246 (4)	151

Symmetry codes: (i) 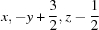; (ii) 
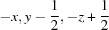
; (iii) 
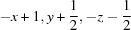
.

**Fig. 1.**
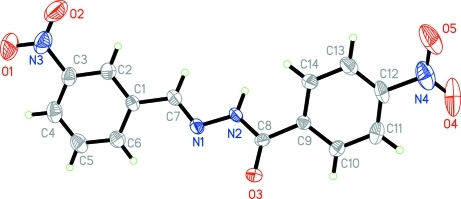
The molecular structure of the title compound, showing the atom labelling scheme. The displacement ellipsoids are drawn at the 30% probability level.

**Fig. 2.**
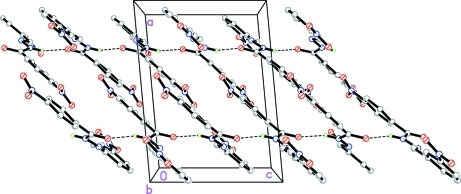
A view along the b axis of the crystal packing of the title compound. The N-H···O hydrogen bonds are indicated by dashed lines (see Table 1 for details). The C-bound H-atoms have been omitted for clarity.

## supplementary crystallographic information

### Comment

In recent years, hydrazone compounds have attracted much attention due to their syntheses and crystal structures (Hashemian *et al.*, 2011; Lei, 2011; Shalash *et al.*, 2010). As a continuation of our work on such compounds (Li, 2011*a*,*b*; Li, 2012), the author reports herein on the crystal structure of the new title hydrazone compound.

The title compound (Fig. 1) exists in an *E* conformation with respect to the C7═N1 bond. The dihedral angle between the (C1–C6) and (C9–C14) benzene rings is 2.41 (14) °.

In the crystal, molecules are linked through N–H···O hydrogen bonds to form chains along the *c* axis (Fig. 2 and Table 1). There are also C-H···O interactions present that link the chains to form a three-dimensional network (Table 1).

### Experimental

A mixture of 3-nitrobenzaldehyde (0.151 g, 1 mmol) and 4-nitrobenzohydrazide (0.181 g, 1 mmol) in 30 ml of ethanol containing few drops of acetic acid was refluxed for about 1 h. On cooling to room temperature, a solid precipitate was formed. The solid was filtered and then recrystallized from methanol. Yellow crystals, suitable for X-ray diffraction analysis, were obtained by slow evaporation of a solution of the title compound in methanol.

### Refinement

The amino H atom was located from a difference Fourier map and was freely refined. The remaining H-atoms were included in calculated positions and refined using a riding model: C–H = 0.93 Å, with *U*_iso_(H) = 1.2*U*_eq_(C).

### Crystal data

**Table:**

C_14_H_10_N_4_O_5_	*F*(000) = 648
*M**_r_* = 314.26	*D*_x_ = 1.452 Mg m^−^^3^
Monoclinic, *P*2_1_/*c*	Mo *K*α radiation, λ = 0.71073 Å
Hall symbol: -P 2ybc	Cell parameters from 979 reflections
*a* = 11.856 (2) Å	θ = 2.3–26.3°
*b* = 14.116 (3) Å	µ = 0.11 mm^−^^1^
*c* = 8.6263 (19) Å	*T* = 298 K
β = 95.193 (2)°	Block, yellow
*V* = 1437.8 (5) Å^3^	0.17 × 0.13 × 0.12 mm
*Z* = 4	

### Data collection

**Table:**

Bruker SMART CCD area-detector diffractometer	2671 independent reflections
Radiation source: fine-focus sealed tube	1288 reflections with *I* > 2σ(*I*)
Graphite monochromator	*R*_int_ = 0.104
ω scans	θ_max_ = 25.5°, θ_min_ = 1.7°
Absorption correction: multi-scan (*SADABS*; Sheldrick, 1996)	*h* = −14→14
*T*_min_ = 0.981, *T*_max_ = 0.987	*k* = −17→16
10319 measured reflections	*l* = −10→10

### Refinement

**Table:**

Refinement on *F*^2^	Primary atom site location: structure-invariant direct methods
Least-squares matrix: full	Secondary atom site location: difference Fourier map
*R*[*F*^2^ > 2σ(*F*^2^)] = 0.052	Hydrogen site location: inferred from neighbouring sites
*wR*(*F*^2^) = 0.123	H atoms treated by a mixture of independent and constrained refinement
*S* = 0.84	*w* = 1/[σ^2^(*F*_o_^2^) + (0.0424*P*)^2^] where *P* = (*F*_o_^2^ + 2*F*_c_^2^)/3
2671 reflections	(Δ/σ)_max_ < 0.001
211 parameters	Δρ_max_ = 0.18 e Å^−^^3^
1 restraint	Δρ_min_ = −0.16 e Å^−^^3^

### Special details

**Table:**

Geometry. Bond distances, angles etc. have been calculated using the rounded fractional coordinates. All su's are estimated from the variances of the (full) variance-covariance matrix. The cell esds are taken into account in the estimation of distances, angles and torsion angles
Refinement. Refinement of *F*^2^ against ALL reflections. The weighted *R*-factor *wR* and goodness of fit *S* are based on *F*^2^, conventional *R*-factors *R* are based on *F*, with *F* set to zero for negative *F*^2^. The threshold expression of *F*^2^ > σ(*F*^2^) is used only for calculating *R*-factors(gt) *etc*. and is not relevant to the choice of reflections for refinement. *R*-factors based on *F*^2^ are statistically about twice as large as those based on *F*, and *R*- factors based on ALL data will be even larger.

### Fractional atomic coordinates and isotropic or equivalent isotropic displacement parameters (Å^2^)

**Table:**

	*x*	*y*	*z*	*U*_iso_*/*U*_eq_	
O1	0.1287 (3)	1.33204 (17)	0.1686 (3)	0.1354 (15)	
O2	0.2448 (3)	1.26884 (18)	0.0245 (4)	0.1188 (15)	
O3	0.23263 (15)	0.65945 (12)	0.1876 (2)	0.0537 (7)	
O4	0.4769 (3)	0.3590 (2)	−0.3204 (4)	0.1556 (18)	
O5	0.5842 (3)	0.4737 (2)	−0.3733 (3)	0.1211 (14)	
N1	0.17622 (18)	0.83659 (16)	0.0848 (2)	0.0491 (8)	
N2	0.22711 (19)	0.76953 (16)	−0.0036 (2)	0.0483 (9)	
N3	0.1702 (3)	1.2632 (2)	0.1105 (4)	0.0899 (16)	
N4	0.5047 (3)	0.4393 (3)	−0.3104 (4)	0.0936 (16)	
C1	0.1348 (2)	1.00191 (19)	0.1080 (3)	0.0425 (10)	
C2	0.1696 (2)	1.0919 (2)	0.0706 (3)	0.0522 (11)	
C3	0.1282 (3)	1.1686 (2)	0.1461 (4)	0.0581 (12)	
C4	0.0505 (3)	1.1592 (2)	0.2540 (4)	0.0726 (14)	
C5	0.0174 (3)	1.0690 (2)	0.2908 (4)	0.0689 (12)	
C6	0.0592 (2)	0.9917 (2)	0.2202 (3)	0.0544 (11)	
C7	0.1814 (2)	0.9203 (2)	0.0303 (3)	0.0486 (10)	
C8	0.2553 (2)	0.68447 (18)	0.0579 (3)	0.0405 (10)	
C9	0.3200 (2)	0.62217 (18)	−0.0417 (3)	0.0387 (9)	
C10	0.3076 (2)	0.52468 (18)	−0.0295 (3)	0.0478 (10)	
C11	0.3684 (3)	0.4655 (2)	−0.1197 (4)	0.0605 (11)	
C12	0.4412 (3)	0.5048 (2)	−0.2157 (3)	0.0608 (11)	
C13	0.4570 (2)	0.5997 (2)	−0.2249 (3)	0.0623 (12)	
C14	0.3961 (2)	0.6581 (2)	−0.1383 (3)	0.0524 (11)	
H2	0.22020	1.10040	−0.00440	0.0630*	
H2A	0.245 (2)	0.7813 (19)	−0.1021 (16)	0.0800*	
H4	0.02120	1.21200	0.30070	0.0870*	
H5	−0.03420	1.06070	0.36470	0.0820*	
H6	0.03660	0.93130	0.24770	0.0650*	
H7	0.21570	0.92980	−0.06130	0.0580*	
H10	0.25910	0.49930	0.03850	0.0570*	
H11	0.35970	0.40020	−0.11490	0.0720*	
H13	0.50850	0.62460	−0.28920	0.0750*	
H14	0.40610	0.72330	−0.14440	0.0630*	

### Atomic displacement parameters (Å^2^)

**Table:**

	*U*^11^	*U*^22^	*U*^33^	*U*^12^	*U*^13^	*U*^23^
O1	0.204 (3)	0.0522 (16)	0.154 (3)	0.0280 (19)	0.039 (2)	−0.0131 (18)
O2	0.128 (3)	0.0639 (18)	0.169 (3)	−0.0092 (17)	0.039 (2)	0.0148 (18)
O3	0.0739 (14)	0.0488 (12)	0.0407 (11)	−0.0012 (10)	0.0178 (10)	0.0052 (10)
O4	0.157 (3)	0.110 (2)	0.196 (4)	0.058 (2)	−0.004 (2)	−0.077 (3)
O5	0.108 (2)	0.174 (3)	0.083 (2)	0.079 (2)	0.0178 (17)	−0.0014 (19)
N1	0.0579 (15)	0.0459 (15)	0.0454 (14)	0.0112 (12)	0.0145 (12)	−0.0004 (12)
N2	0.0655 (17)	0.0451 (14)	0.0371 (14)	0.0116 (12)	0.0198 (12)	0.0010 (12)
N3	0.116 (3)	0.051 (2)	0.101 (3)	0.012 (2)	0.000 (2)	0.0029 (19)
N4	0.080 (3)	0.125 (3)	0.072 (2)	0.054 (3)	−0.0140 (19)	−0.019 (2)
C1	0.0389 (16)	0.0469 (18)	0.0411 (16)	0.0061 (14)	0.0011 (13)	−0.0036 (14)
C2	0.0483 (19)	0.0519 (19)	0.0563 (19)	0.0063 (15)	0.0035 (15)	0.0006 (15)
C3	0.067 (2)	0.045 (2)	0.061 (2)	0.0126 (17)	−0.0010 (17)	−0.0018 (16)
C4	0.082 (3)	0.062 (2)	0.073 (2)	0.022 (2)	0.002 (2)	−0.0110 (19)
C5	0.061 (2)	0.079 (2)	0.068 (2)	0.022 (2)	0.0138 (17)	−0.005 (2)
C6	0.0494 (18)	0.058 (2)	0.0562 (19)	0.0044 (15)	0.0064 (15)	−0.0017 (16)
C7	0.0488 (18)	0.0550 (19)	0.0426 (17)	0.0011 (15)	0.0078 (14)	−0.0057 (15)
C8	0.0452 (17)	0.0394 (17)	0.0374 (16)	−0.0084 (13)	0.0061 (13)	0.0025 (13)
C9	0.0395 (16)	0.0402 (16)	0.0365 (15)	0.0007 (13)	0.0038 (13)	0.0013 (13)
C10	0.0509 (18)	0.0423 (17)	0.0494 (18)	−0.0025 (15)	0.0000 (14)	0.0004 (14)
C11	0.066 (2)	0.0439 (18)	0.067 (2)	0.0093 (17)	−0.0183 (18)	−0.0127 (17)
C12	0.059 (2)	0.074 (2)	0.0479 (19)	0.0325 (19)	−0.0027 (16)	−0.0147 (18)
C13	0.059 (2)	0.076 (2)	0.054 (2)	0.0204 (18)	0.0167 (17)	0.0064 (18)
C14	0.0540 (19)	0.0489 (18)	0.0566 (19)	0.0053 (15)	0.0176 (15)	0.0063 (15)

### Geometric parameters (Å, º)

**Table:**

O1—N3	1.218 (4)	C5—C6	1.365 (4)
O2—N3	1.207 (5)	C8—C9	1.490 (4)
O3—C8	1.226 (3)	C9—C14	1.379 (4)
O4—N4	1.181 (5)	C9—C10	1.389 (4)
O5—N4	1.229 (5)	C10—C11	1.387 (4)
N1—N2	1.387 (3)	C11—C12	1.367 (5)
N1—C7	1.275 (4)	C12—C13	1.356 (4)
N2—C8	1.343 (3)	C13—C14	1.363 (4)
N3—C3	1.467 (4)	C2—H2	0.9300
N4—C12	1.483 (5)	C4—H4	0.9300
N2—H2A	0.910 (16)	C5—H5	0.9300
C1—C6	1.385 (4)	C6—H6	0.9300
C1—C7	1.466 (4)	C7—H7	0.9300
C1—C2	1.383 (4)	C10—H10	0.9300
C2—C3	1.377 (4)	C11—H11	0.9300
C3—C4	1.374 (5)	C13—H13	0.9300
C4—C5	1.378 (4)	C14—H14	0.9300
			
N2—N1—C7	113.1 (2)	C8—C9—C14	122.0 (2)
N1—N2—C8	119.87 (19)	C9—C10—C11	119.4 (2)
O1—N3—O2	123.1 (3)	C10—C11—C12	119.0 (3)
O1—N3—C3	118.9 (3)	N4—C12—C13	120.3 (3)
O2—N3—C3	118.0 (3)	N4—C12—C11	117.4 (3)
O4—N4—O5	124.5 (4)	C11—C12—C13	122.3 (3)
O4—N4—C12	119.1 (3)	C12—C13—C14	118.9 (3)
O5—N4—C12	116.4 (4)	C9—C14—C13	121.1 (3)
C8—N2—H2A	117.5 (17)	C1—C2—H2	120.00
N1—N2—H2A	122.6 (17)	C3—C2—H2	121.00
C2—C1—C7	118.9 (2)	C3—C4—H4	121.00
C2—C1—C6	119.0 (2)	C5—C4—H4	121.00
C6—C1—C7	122.2 (2)	C4—C5—H5	120.00
C1—C2—C3	119.0 (2)	C6—C5—H5	120.00
N3—C3—C4	119.4 (3)	C1—C6—H6	120.00
N3—C3—C2	118.3 (3)	C5—C6—H6	120.00
C2—C3—C4	122.3 (3)	N1—C7—H7	119.00
C3—C4—C5	117.9 (3)	C1—C7—H7	119.00
C4—C5—C6	120.9 (3)	C9—C10—H10	120.00
C1—C6—C5	120.9 (3)	C11—C10—H10	120.00
N1—C7—C1	121.8 (2)	C10—C11—H11	120.00
O3—C8—C9	121.5 (2)	C12—C11—H11	121.00
N2—C8—C9	115.0 (2)	C12—C13—H13	121.00
O3—C8—N2	123.4 (2)	C14—C13—H13	120.00
C10—C9—C14	119.3 (2)	C9—C14—H14	119.00
C8—C9—C10	118.5 (2)	C13—C14—H14	119.00
			
C7—N1—N2—C8	162.4 (2)	C1—C2—C3—C4	−2.2 (5)
N2—N1—C7—C1	−178.7 (2)	N3—C3—C4—C5	−176.6 (3)
N1—N2—C8—O3	4.4 (4)	C2—C3—C4—C5	2.6 (5)
N1—N2—C8—C9	−173.9 (2)	C3—C4—C5—C6	−1.1 (5)
O1—N3—C3—C2	175.1 (3)	C4—C5—C6—C1	−0.9 (5)
O1—N3—C3—C4	−5.6 (5)	O3—C8—C9—C10	31.6 (4)
O2—N3—C3—C2	−4.9 (5)	O3—C8—C9—C14	−144.7 (3)
O2—N3—C3—C4	174.3 (4)	N2—C8—C9—C10	−150.0 (2)
O4—N4—C12—C11	12.8 (5)	N2—C8—C9—C14	33.7 (3)
O4—N4—C12—C13	−167.8 (3)	C8—C9—C10—C11	−179.1 (3)
O5—N4—C12—C11	−166.9 (3)	C14—C9—C10—C11	−2.7 (4)
O5—N4—C12—C13	12.5 (5)	C8—C9—C14—C13	178.0 (2)
C6—C1—C2—C3	0.1 (4)	C10—C9—C14—C13	1.8 (4)
C7—C1—C2—C3	−178.4 (3)	C9—C10—C11—C12	1.6 (4)
C2—C1—C6—C5	1.4 (4)	C10—C11—C12—N4	−180.0 (3)
C7—C1—C6—C5	179.9 (3)	C10—C11—C12—C13	0.7 (5)
C2—C1—C7—N1	162.1 (2)	N4—C12—C13—C14	179.0 (3)
C6—C1—C7—N1	−16.4 (4)	C11—C12—C13—C14	−1.7 (4)
C1—C2—C3—N3	177.1 (3)	C12—C13—C14—C9	0.4 (4)

### Hydrogen-bond geometry (Å, º)

**Table:**

*D*—H···*A*	*D*—H	H···*A*	*D*···*A*	*D*—H···*A*
N2—H2*A*···O3^i^	0.91 (2)	1.99 (2)	2.853 (3)	158 (2)
C6—H6···O1^ii^	0.93	2.57	3.369 (4)	145
C7—H7···O5^iii^	0.93	2.56	3.287 (4)	135
C7—H7···O3^i^	0.93	2.53	3.271 (3)	137
C14—H14···O4^iii^	0.93	2.40	3.246 (4)	151

Symmetry codes: (i) *x*, −*y*+3/2, *z*−1/2; (ii) −*x*, *y*−1/2, −*z*+1/2; (iii) −*x*+1, *y*+1/2, −*z*−1/2.
